# Protein, Nucleic Acid, and Nanomaterial Engineering for Biosensors and Monitoring

**DOI:** 10.3390/bios15070430

**Published:** 2025-07-03

**Authors:** Milica Crnoglavac Popović, Vesna Stanković, Dalibor Stanković, Radivoje Prodanović

**Affiliations:** 1 Faculty of Chemistry, University of Belgrade, Studentski trg 12, 11000 Belgrade, Serbia; milicac@chem.bg.ac.rs (M.C.P.); dalibors@chem.bg.ac.rs (D.S.); 2 IHTM, Njegoševa 12, 11000 Belgrade, Serbia; vesna.stankovic@ihtm.bg.ac.rs

**Keywords:** directed evolution, DNA, RNA, nanocomposites, MOF, biosensor, rare diseases

## Abstract

The engineering of proteins, nucleic acids, and nanomaterials has significantly advanced the development of biosensors for the monitoring of rare diseases. These innovative biosensing technologies facilitate the early detection and management of conditions that often lack adequate diagnostic solutions. By utilizing engineered proteins and functional nucleic acids, such as aptamers and nucleic acid sensors, these biosensors can achieve high specificity in identifying the biomarkers associated with rare diseases. The incorporation of nanomaterials, like nanoparticles and nanosensors, enhances sensitivity and allows for the real-time monitoring of biochemical changes, which is critical for timely intervention. Moreover, integrating these technologies into wearable devices provides patients and healthcare providers with continuous monitoring capabilities, transforming the landscape of healthcare for rare diseases. The ability to detect low-abundance biomarkers in varied sample types, such as blood or saliva, can lead to breakthroughs in understanding disease pathways and personalizing treatment strategies. As the field continues to evolve, the combination of protein, nucleic acid, and nanomaterial engineering will play a crucial role in developing next-generation biosensors that are not only cost-effective but also easy to use, ultimately improving outcomes and the quality of life for individuals affected by rare diseases.

## 1. Introduction

**Figure 1:**
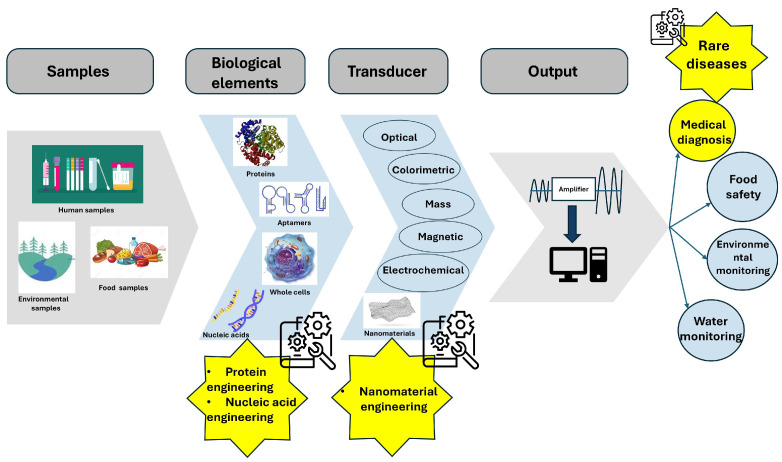
Biomolecular and nanomaterial engineering in the development of biosensors for monitoring.

In this review, we will cover the most recent studies concerning protein, nucleic acid, and nanomaterial engineering for biosensors that can detect and be used for the monitoring of rare diseases, as detailed in Figure 1.

The rapid advancement of biosensor technology has emerged as a transformative force in diagnostics, enabling the detection of rare diseases through the engineering of proteins, nucleic acids, and nanomaterials. Rare diseases often present significant challenges in terms of diagnosis, given the complexity and low prevalence of the associated biomarkers. To address these challenges, this review focuses on the engineering strategies applied to enhance the performance of biosensors, thus facilitating the timely detection and monitoring of rare illnesses.

The optical and electrochemical biosensors discussed in this article operate on distinct, yet complementary, principles for detecting biological interactions. Optical biosensors detect target analytes by measuring changes in optical properties, such as absorbance, fluorescence, luminescence, or the refractive index, resulting from biomolecular recognition events on the sensor’s surface. Techniques like surface plasmon resonance (SPR), fluorescence spectroscopy, and interferometry are commonly employed to monitor these changes, often enabling label-free and real-time analyses with high sensitivity. In contrast, electrochemical biosensors convert biochemical interactions into electrical signals—such as current, voltage, or impedance—using recognition elements, like enzymes, antibodies, or aptamers. Amperometric, potentiometric, and impedimetric approaches are used to quantify these signals, offering advantages such as a low cost, portability, and suitability for point-of-care diagnostics.

Section 2 of this review addresses protein engineering for biosensors, including directed evolution, semi-rational design, and rational design approaches, each contributing to the development of highly targeted biosensors with improved binding capabilities. In Section 3, we further explore the engineering of nucleic acids, particularly RNA and DNA, which have revolutionized biosensor specificity and sensitivity by developing novel aptamers and genetic circuits.

Section 4 discusses the engineering of nanomaterials and highlights their critical role in amplifying sensor signals and improving the overall biosensor performance. Finally, Section 5 examines the application of these engineered biosensors for monitoring rare diseases, emphasizing their potential impact on personalized medicine and patient outcomes. Through this synthesis, the review emphasizes the critical intersections of these fields, paving the way for future advancements in biosensing technologies to enhance the management of rare diseases.

## 2. Protein Engineering for Biosensors

### 2.1. Directed Evolution of Proteins for Biosensors

Directed evolution has rapidly emerged as a transformative strategy for developing biosensor proteins, enabling researchers to create highly tailored biomolecules that respond to specific analytes with improved sensitivity and selectivity. By mimicking natural selection principles through iterative mutation, selection, and amplification rounds, scientists can evolve proteins that outperform their natural counterparts. For instance, in the development of biosensors for detecting the R248Q mutant of the tumor suppressor protein p53, Pellerano et al. effectively utilized directed evolution to generate a fluorescent peptide biosensor that demonstrated a significant interaction with the aggregation-prone variant of p53, showing the potential of this strategy in clinical applications and biomarker detection [1]. This approach highlights how engineered proteins can provide critical insights into specific mutations linked to disease states.

**Figure 2:**
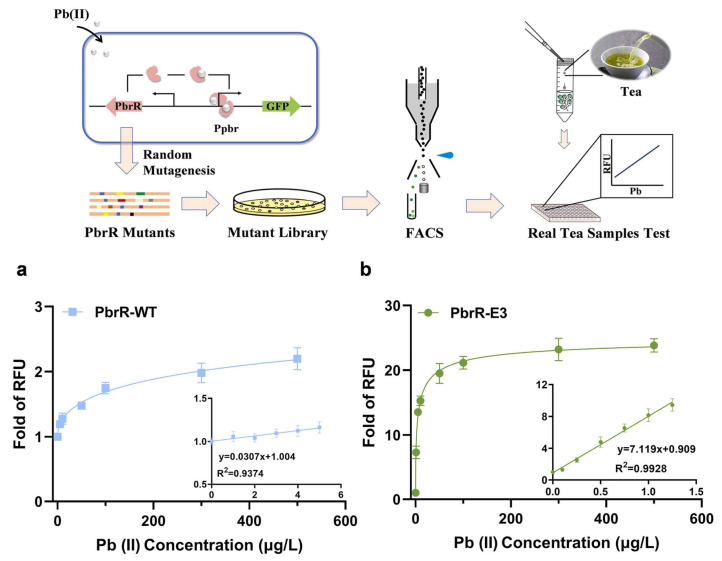
A strategy for the development of a highly sensitive, PbrR-based biosensor via directed evolution and its application for lead detection. (**a**) The dose-dependent response of PbrR-WT WCB detected by flow cytometer. (**b**) The dose-dependent response of PbrR-E3 WCB detected by flow cytometer. Reprinted from ref. [2] with permission. Copyright © 2025 Elsevier B.V.

Additionally, Liang et al. developed a lead (Pb) whole-cell biosensor based on the Pb resistance transcriptional regulatory factor and green fluorescent protein and improved its performance through directed evolution in conjunction with fluorescence-activated cell sorting (FACS) [2], Figure 2.

**Figure 3:**
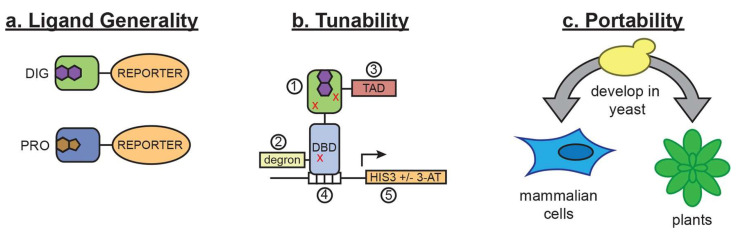
A schematic of the biosensor platform. (**a**) Biosensors for small molecules are modularly constructed by replacing the LBD with proteins possessing altered substrate preferences. (**b**) The activity of the biosensor can be tuned by (1) introducing destabilizing mutations (red Xs), (2) adding a degron, (3) altering the strength of the TAD or DNA binding affinity of the TF, (4) changing the number of TF-binding sites or sequences, and (5) titrating 3-aminotriazole, an inhibitor of His3. (**c**) Yeast provides a genetically tractable chassis for biosensor development before implementation in more complex eukaryotes, such as mammalian cells and plants. Reprinted from ref. [3]; licensed under a Creative Commons Attribution license (CC-BY).

The adaptability of directed evolution is further illustrated by its utilization in the development of transcription-factor-based biosensors, which are highly effective in monitoring intricate biological systems. Feng et al. [3] proposed a comprehensive framework for the construction of small-molecule biosensors within eukaryotic contexts, utilizing a ligand-binding domain (LBD) fused to a transcriptional activator, Figure 3.

In this system, the binding of the ligand serves to stabilize the LBD, thereby preventing its degradation. By fusing this destabilized LBD to an appropriate reporter protein—such as an enzyme, a fluorescent protein, or a transcription factor—the resulting construct becomes conditionally stable and produces a measurable sensor response. When conditionally stabilized LBDs are linked to transcription factors (TFs), the resulting biosensors exhibit a higher degree of signal induction in response to target ligands compared to those fused with fluorescent proteins. Utilizing these TF-biosensors, the authors successfully enhanced the biosynthetic yield of progesterone in yeast, demonstrating the practical applications of this biosensing technology [3]. Researchers can create biosensors with the precise response characteristics needed for various small molecules or environmental conditions by systematically mutating transcription factors and utilizing selection systems.

In a study by Paulmurugan et al. [4], a novel protein folding molecular imaging biosensor is introduced to monitor the drug effects that restore the structure and function of the mutant p53 protein in glioblastoma cells. This innovative assay enables rapid high-throughput screening for compounds that promote proper p53 folding, with potential applications for non-invasive studies in living subjects using optical bioluminescence imaging in small animal models. The research highlights the biosensor’s effectiveness in screening drugs that reactivate p53 activity, representing a promising advancement in cancer therapy targeting p53 mutations. This technique not only streamlines the biosensor design process but also accelerates the timeline from concept to functioning sensor in dynamic biological systems.

Additionally, integrating novel strategies, such as Förster resonance energy transfer (FRET), into directed evolution allows for a more refined biosensor architecture that can yield more nuanced insights into biological processes. In the publication by Taylor et al. [5] a significant advancement in protein engineering is demonstrated through the development of allosteric transcription factors (aTFs) that can be tailored to respond to novel inducers. The authors employed the *Escherichia coli* lac repressor, LacI, and modified its structure to achieve responsiveness to new small molecules, such as fucose and gentiobiose. By integrating computational design with directed mutagenesis techniques, they successfully generated LacI variants that maintain specificity and induction efficiencies comparable to the native protein when activated by the traditional inducer IPTG. This engineering strategy not only enables the precise control of gene expression in synthetic biology applications but also opens up avenues for designing biosensors capable of detecting specific biomolecules in complex environments. The ability to create customized biosensors based on engineered proteins represents a significant leap forward in both biosensing technology and metabolic engineering, enabling the monitoring and manipulation of cellular processes with unprecedented specificity and sensitivity.

Furthermore, in a study by Gohil et al. [6] the authors present a comprehensive evaluation of biosensor optimization utilizing Förster resonance energy transfer (FRET) pairs based on the mScarlet red fluorescent protein and a derived green fluorescent protein. The research explores two primary classes of biosensors: one that employs intensiometric measurements, where a conformational change in the recognition domain is relayed to a single fused fluorescent protein, resulting in altered fluorescence intensity; and another that utilizes the recognition-domain-dependent modulation of FRET between two fluorescent proteins of differing colors, allowing for ratiometric changes in fluorescence emission. The optimization strategies detailed in the study aim to enhance the sensitivity and specificity of FRET-based biosensors, which have been successfully engineered to monitor various intracellular processes, including enzyme activity, ion dynamics, post-translational modifications, and protein–protein interactions. This work highlights the potential of utilizing engineered fluorescent protein pairs to develop advanced biosensors that provide real-time insights into complex biological systems, thereby facilitating a deeper understanding of cellular dynamics and interactions.

**Figure 4:**
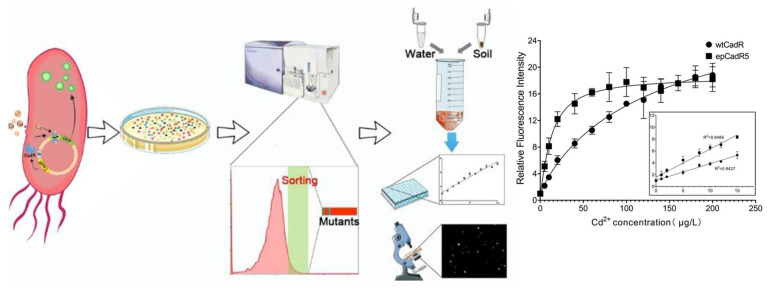
Strategy for evolving bacterial biosensor for measuring cadmium concentration. Dose-dependent response of bacterial biosensors to cadmium in *E. coli* strain JudeI. Reprinted from ref. [7] with permission. Copyright © 2022 American Chemical Society.

Cai et al. [7] developed an advanced biosensor for the sensitive and specific detection of cadmium in environmental samples by integrating an engineered cadmium resistance transcriptional regulator (CadR) with a green fluorescent protein (GFP) reporter, Figure 4.

Using directed evolution, the researchers generated mutant libraries via error-prone PCR and optimized the biosensor through multiple rounds of fluorescence-activated cell sorting (FACS). This process led to the identification of the variant epCadR5, which exhibited significantly improved performance. The evolved biosensor achieved a remarkable detection limit of 0.45 μg/L for cadmium, demonstrating 6.8 times greater specificity compared to the wild-type sensor. This study highlights the potential of directed evolution in enhancing biosensor functionality for environmental monitoring, thereby facilitating the more reliable detection of hazardous heavy metals, such as cadmium.

Guo et al. [8] explored the potential of using the anthocyanin biosynthetic pathway to develop a bacterial biosensor for detecting lead toxicity. By engineering the metalloregulator PbrR to regulate anthocyanin biosynthesis in response to lead (Pb) levels, the researchers created a visually detectable biosensor that changes color upon exposure to this toxic heavy metal. Their findings highlight the effectiveness of metabolic engineering in utilizing natural pigments for the development of sensitive, cost-effective biosensors for monitoring environmental toxins. This approach highlights the ecological significance of tracking lead pollution and demonstrates the potential of biosensors in assessing the bioavailability and ecotoxicity of heavy metals. Overall, the study represents a significant advancement in biosensing technology, contributing to the development of practical tools for environmental monitoring. This application extends beyond basic detection; it aims to identify potential toxicity issues in aquatic ecosystems early, thereby emphasizing the societal relevance of such biosensor technologies.

In a study by Lee et al. [9], the authors explore the development and application of genetic enzyme screening systems (GESSs) specifically designed for the detection of C1 compounds—formate, formaldehyde, and methanol—using genetically engineered biosensors. The research presents the FA-GESS, Frm-GESS, and MeOH-GESS biosensors, which incorporate enzymatic pathways that enable the specific detection of these compounds through their respective enzymatic activities. Each biosensor demonstrates a linear response to its target compound—formate (1.0–250 mM), formaldehyde (1.0–50 μM), and methanol (5–400 mM)—showing the high specificity that is crucial for practical applications. By harnessing the capabilities of bacterial oxidoreductases within these biosensing techniques, the study significantly broadens the spectrum of detectable ligands available to genetic biosensor systems. Overall, their work underlines the potential of engineered biosensors in environmental monitoring and metabolic engineering, offering valuable tools for the real-time analysis of key C1 pollutants.

Fana et al. [10] applied directed evolution to enhance the thermostability of cholesterol oxidase (ChOx), a key enzyme in cholesterol biosensors. Using error-prone PCR, they generated a diverse library of ChOx variants and identified mutants with improved stability, crucial for maintaining biosensor reliability in dynamic environments. Their study underscores the power of directed evolution in enzyme engineering, demonstrating its potential to create more sensitive and durable biosensors for cholesterol monitoring. By optimizing ChOx, the research advances medical diagnostics and metabolic monitoring, highlighting the critical role of engineered enzymes in biosensor development.

Despite the successes observed in the directed evolution of proteins for biosensing applications, challenges remain in optimizing the protein-engineering process to meet specific assay requirements. The increasing complexity of biological systems necessitates continuously refining biosensors to ensure robust performance under variable conditions. Current research indicates that a combination of computational modeling alongside empirical evolution techniques will be crucial for future research in protein engineering for biosensors. This approach will enable biosensor development and advance our understanding of the underlying molecular dynamics that govern protein–analyte interactions.

### 2.2. Semi-Rational Design of Proteins for Biosensors

The semi-rational design of proteins for biosensors combines computational modeling and empirical experimentation to optimize the performance of these biological sensors. This strategy enables targeted modifications of proteins or receptor elements, leveraging structural information to enhance binding affinity and specificity toward analytes. For example, Ding et al. used a semi-rational approach to fine-tune cross-ribosome-binding sites in a glucarate biosensor, significantly improving its dynamic range [11]. It represents a rational design strategy for fine-tuning the dynamic range of bacterial biosensors by engineering cross-ribosome-binding sites. The study focuses on optimizing biosensor performance for detecting specific target molecules by precisely adjusting expression levels, enabling a more controlled response over a broader concentration range. By strategically modifying ribosome-binding sites, the researchers enhance the balance between sensitivity and specificity, thereby improving the applicability of biosensors in fields such as environmental monitoring and metabolic engineering. Their findings demonstrate the potential of this approach to enhance biosensor efficacy and practicality, offering a framework for refining existing biosensors and guiding the development of next-generation biosensing technologies with tailored response characteristics. Such methodologies illustrate that semi-rational design can efficiently utilize existing biological and computational knowledge to create more-effective biosensors, making it a valuable tool in synthetic biology and biotechnological applications.

**Figure 5:**
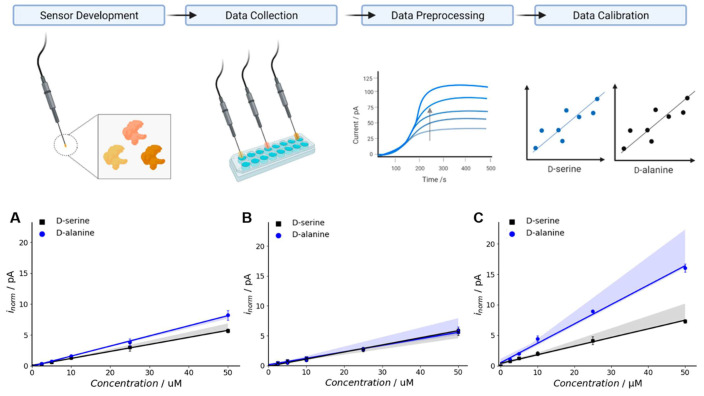
Strategy for enhancing electrochemical biosensor selectivity with engineered d-amino acid oxidase enzymes for d-serine and d-alanine quantification. RgDAAO WT (**A**), RgDAAO M213G (**B**), and hDAAO W209R (**C**) biosensor calibration in standard solutions of 0–50 μM d-serine and d-alanine. Error bars represent ± S.E.M, (*n* = 3). The shaded areas represent 95% confidence interval regions. Reprinted from ref. [12] with permission. Copyright © 2022 American Chemical Society.

In addition to improving binding affinities, semi-rational design strategies have been employed to enhance the selectivity of biosensors for complex mixtures. Moussa et al. [12] reported engineering d-amino acid oxidase enzymes to increase their selectivity for d-serine and d-alanine, demonstrating the potential for the more sensitive detection of these amino acids in biological samples, Figure 5.

Given the crucial roles of these d-amino acids in neurotransmission and metabolic regulation, accurate detection is essential for biomedical research. Using targeted mutagenesis, the authors developed enzyme variants with enhanced catalytic efficiency and substrate specificity, enabling the selective detection of targets in complex biological matrices. The resulting electrochemical biosensor exhibited a low limit of detection and quantification, making it a valuable tool for applications in neuroscience and metabolic studies. The study employed rational design techniques to introduce point mutations in the DAAO enzyme, which enhance its substrate specificity. The two engineered variants, human DAAO W209R and yeast DAAO M213G, exhibit significant improvements in selectivity and activity compared to the wild-type enzyme. The authors immobilized these variants to facilitate practical biosensor application, demonstrating that the resulting electrochemical biosensors achieve selectivity levels comparable to those observed with free DAAO enzymatic activity. Their findings emphasize the importance of integrating engineered biocatalysts into biosensor design to enhance performance in terms of selectivity and sensitivity. It also highlights the potential for creating sensitive tools for measuring D-amino acids, which are crucial in various biological processes and hold promise for potential therapeutic applications.

Furthermore, as semi-rational design continues to evolve, integrating high-throughput screening techniques facilitates the rapid evaluation of protein variants, identifying optimal candidates for biosensing applications. Approaches like domain-insertion profiling, as employed by Nadler et al., rapidly construct metabolite biosensors by assessing diverse protein libraries with high-throughput assays [13]. The authors introduce a methodology for the rapid development of single-fluorescent protein biosensors (SFPBs) designed to detect specific metabolites in living cells. Traditional biosensor design has been hindered by the challenge of achieving effective allosteric coupling between a green fluorescent protein (GFP) and a ligand-binding domain, limiting biosensor efficiency and functionality. To overcome this limitation, the researchers employed domain-insertion profiling, a systematic strategy that accelerates the identification of optimal insertion sites within the ligand-binding domain, streamlining the biosensor engineering process. The resulting SFPBs demonstrated exceptional performance, enabling the real-time quantification of metabolites, such as trehalose, at the single-cell level. Their study represents a significant advancement in biosensing technology, offering a scalable and efficient approach for developing high-performance biosensors applicable to diverse biological and medical contexts, ultimately improving our ability to monitor metabolite dynamics in complex environments. This integration of computational predictions and experimental datasets supports the dynamic profiling of protein-based biosensors.

Zhou et al. [14] described a novel approach to enhancing metabolite biosensing in *Saccharomyces cerevisiae* by integrating DNA barcoding with machine learning techniques. Their study focused on constructing a biosensor system using six distinct promoters (pGPD, pENO2, pHSP12, pEXG1, pCYC1, pULI1) with varying activity levels to monitor biochemical reactions based on transcription factor (TF) dosages. By employing a minimal synthetic promoter with a shortened sequence length, high robustness, and strong orthogonality, the authors drove the expression of the fluorescent protein YPet. A key challenge in biosensor development is gene expression noise at the single-cell level, arising from both extrinsic factors, such as cell morphology, plasmid copy number, and cell cycle stage, and intrinsic factors, including fluctuations in DNA, RNA, enzymes, and TF levels. The researchers addressed this issue by integrating machine learning into response curve profiling, which enables the improved deconvolution of noise factors and a more precise characterization of biosensor performance. Their study improves biosensing technology by demonstrating how computational approaches can enhance the accuracy and reliability of biosensors. Using machine learning for data-driven optimization, the work provides a way to develop more sophisticated and high-precision biosensors.

Jiang et al. [15] enhanced the thermostability of *Culex pipiens* acetylcholinesterase (AChE) using a semi-rational design approach, combining computational modeling and site-directed mutagenesis to identify stabilizing mutations. The engineered AChE exhibits improved thermal stability, making it more suitable for biosensing applications. Additionally, a sensitivity analysis confirmed its effectiveness in detecting acephate, an organophosphate pesticide, highlighting its potential for environmental monitoring. This study advances enzyme engineering for robust biosensors capable of detecting environmental contaminants under challenging conditions.

Balaž et al. [16] enhanced the stability of cellobiose dehydrogenase (CDH) using a semi-rational design approach, improving its resilience to peroxide, a common inhibitor in biological systems. By integrating structural insights with targeted mutagenesis, they identified key mutations that significantly increased the enzyme’s thermal stability and oxidative resistance. Three mutants with increased activity and stability in the presence of peroxide were found: M65F, M738S, and M685Y. These advancements are particularly valuable for biosensors that detect carbohydrates in complex samples, ensuring reliable performance under challenging conditions. This study highlights the potential of targeted enzyme engineering to optimize biocatalysts for biosensing applications, driving progress in biotechnology and environmental monitoring.

Collectively, the semi-rational design of proteins serves as the methodology of choice in improving biosensors that should meet the increasing demands in medical diagnostics, environmental monitoring, and synthetic biology [17].

### 2.3. Rational Design of Proteins for Biosensors

The rational design of proteins for biosensors is a sophisticated approach that uses an understanding of protein structure and function to optimize performance in biosensing applications. This method involves the precise engineering of protein scaffolds to enhance their binding affinity and specificity for target analytes, thereby improving the sensitivity of biosensors.

For instance, Pedotti et al. demonstrated the rational modification of estrogen receptor proteins to detect endocrine disruptors (EDCs) with increased sensitivity and specificity, showcasing the potential for tailored receptor designs in environmental monitoring [18]. It represents an innovative strategy for developing biosensing techniques to detect endocrine-disrupting chemicals (EDCs) in environmental samples by leveraging rationally engineered estrogen receptor (ER) proteins as biorecognition elements. Through protein engineering, the researchers designed recombinant ER variants with enhanced binding affinities for specific EDCs, significantly improving assay sensitivity and specificity compared to traditional detection methods. These engineered receptors enable the detection of estradiol, a well-characterized EDC, and offer a more sensitive and convenient alternative to conventional mass spectrometry techniques. This study highlights the potential of modifying natural protein receptors to enhance biosensor performance, opening new avenues for environmental monitoring and pollutant detection using biosensing technology.

Integrating computational modeling with empirical data enhances this design process, enabling researchers to predict how changes in amino acid sequences affect protein interactions and activity, thereby guiding the rational design effort.

This level of control is crucial for applications that require the precise quantification of metabolites or experimental conditions. Additionally, synthetic biosensors have been developed to monitor metabolic states in real-time by engineering transcription factors that respond to specific ligands, as illustrated by Rogers et al. [19], who explored transcription-factor-based synthetic biosensors for precise gene regulation and real-time metabolite monitoring in microbial and mammalian systems. Luminescent, protein-based sensors enable dynamic transcriptional control in response to environmental or intracellular signals. Key regulators, such as MphR (responsive to macrolide antibiotics) and TtgR (modulating multidrug efflux pumps via flavonoids), demonstrate broad applicability in gene expression modulation. This study highlights the potential of synthetic biology to enhance biosensing technology, advancing real-time metabolic monitoring and genetic control in diverse research and medical fields.

Hiraka et al. [20] optimized *Aerococcus viridans* l-lactate oxidase (LOX) using rational design to enhance lactate biosensor performance by facilitating quasi-direct electron transfer. Through targeted mutations, Ala96Leu and Asn212Lys, they enhanced the lactate binding affinity and electron transfer efficiency, resulting in increased sensitivity and a lower detection limit, critical for medical applications, such as lactic acid monitoring in patients. The study highlights the potential of enzyme engineering in biosensor development and underscores the role of immobilization techniques in enhancing stability and reproducibility, advancing clinical and biotechnological biosensing technologies.

Golynskiy et al. emphasized the utility of engineered protein switches that translate biochemical signals into measurable outputs, a key attribute for developing efficient biosensing systems [21]. This trend is further supported by efforts to create multicolored biosensors through synthetic biology techniques, which enable high-throughput and multiparametric analyses of biological systems.

Moreover, ongoing research highlights the importance of incorporating advanced techniques, such as Förster resonance energy transfer (FRET) and the engineering of allosteric proteins, into the rational design of biosensors. Hellweg et al. [22] for example, address the limitations of traditional fluorescent biosensors by developing a novel family of FRET pairs with near-quantitative efficiencies. Utilizing reversible interactions between fluorescent proteins and a labeled HaloTag, this approach enables biosensors with significantly expanded dynamic ranges. The engineered FRET pairs enable the precise monitoring of key cellular metabolites, such as calcium, ATP, and NAD+, while facilitating multicolor, simultaneous imaging in live cells. This method enhances biosensor versatility, advancing research on metabolic processes and cellular signaling with greater accuracy and sensitivity.

For instance, Kimura et al. [23] demonstrated this technique by evolving the LuxR protein to modify its responsiveness to signaling molecules, resulting in a more stringent biosensor capable of detecting lower concentrations of target analytes.

Similarly, directed evolution strategies have been employed to fine-tune the functionalities of transcription factors, resulting in significant improvements in the recognition of environmental pollutants and biological markers. The ability to systematically adjust biosensor properties makes directed evolution a powerful tool in biosensor engineering.

In a study by Du et al., the researchers explored the engineering of biosensors responsive to 4-hydroxyphenylpyruvate (HPP) using a novel dual-selection system. They employed the transcription factor PobR from *Acinetobacter* ADP1, which activates the PpobA promoter upon binding its natural ligand, 4-hydroxybenzoic acid (4HB). The aim was to create a biosensor capable of detecting HPP by generating mutant variants of PobR that are specifically responsive to this substrate. By implementing a selection protocol in *Escherichia coli*, the study successfully identified mutant PobR variants that exhibit enhanced sensitivity and specificity toward HPP, thereby demonstrating the potential of directed evolution and rational design strategies to optimize biosensor performance and expand their applicability in synthetic biology [24].

**Figure 6:**
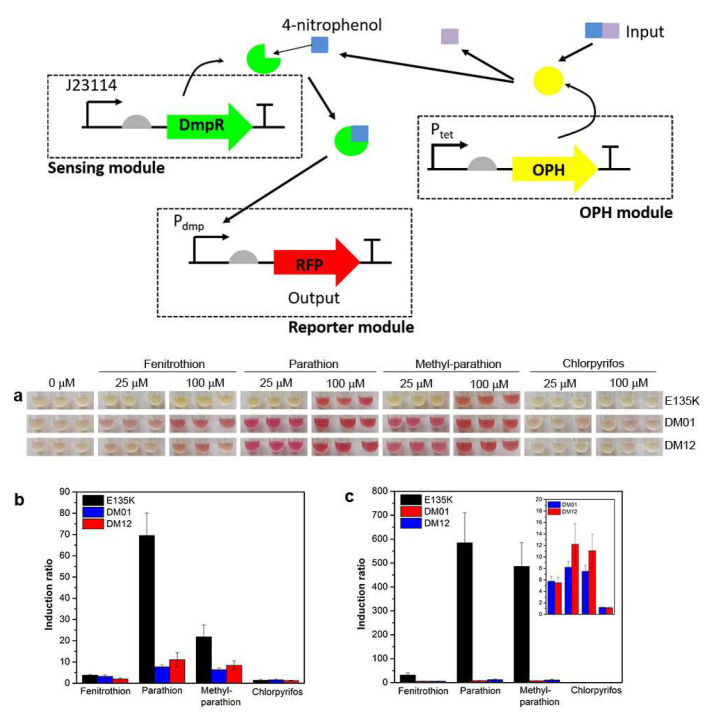
Strategy for development of colorimetric-based, whole-cell biosensor for organophosphorus compounds by engineering transcription regulator DmpR. (**a**) Color of liquid cultures of DmpR mutants after overnight exposure to various OP pesticides (fenitrothion, parathion, methyl-parathion, and chlorpyrifos) at concentrations of 25 and 100 μM at 30 °C at 225 rpm. (**b**) Induction ratios of DmpR mutants after overnight induction to 25 μM OP pesticides at 30 °C at 225 rpm. (**c**) Induction ratios of DmpR mutants after overnight induction to 100 μM OP pesticides at 30 °C at 225 rpm. Reprinted from ref. [25] with permission. Copyright © 2022 American Chemical Society.

In their 2016 study, Chong and Ching focused on the development of a colorimetric-based, whole-cell biosensor specifically designed to detect organophosphorus compounds, such as those found in pesticides, Figure 6 [25].

They employed directed evolution techniques on the transcription regulator DmpR to optimize the system’s responsiveness to these compounds. By modifying the DmpR regulator, they aimed to enhance the expression of a promoter fused to the red fluorescent protein (mRFP1), enabling a visual red coloration as an output signal upon detecting organophosphate pesticides with a phenolic group. The researchers addressed the inherent limitation of colorimetric biosensors, which typically exhibit higher detection limits compared to fluorescence or bioluminescence methods. Their results demonstrated improved sensitivity and effectiveness in utilizing colorimetric signals for detecting hazardous substances, thereby advancing the field of biosensing technology in environmental monitoring [25].

As biotechnological applications grow, the rational design of proteins for biosensors will drive significant innovations in detecting and quantifying various biological targets, enhancing our capabilities across biomedicine and environmental science.

## 3. Nucleic Acid Engineering for Biosensors

Nucleic acids can be utilized as biosensors to analyze biomarkers within living cells, which is crucial for understanding cellular dynamics and achieving accurate disease diagnoses. These biosensors utilize specific DNA sequences as probes to detect a wide range of biomarkers, providing significant insights for disease diagnostics and drug development. As these methodologies continue to evolve, they will expand the capabilities of DNA biosensors, making them indispensable for applications in disease diagnostics, environmental monitoring, and biotechnology [26,27].

The combination of directed evolution with state-of-the-art screening methods promises to drive innovation in biosensor technology, making it a vibrant area of research.

Directed evolution has emerged as a powerful strategy in the field of RNA engineering, particularly for the development of biosensors with enhanced performance characteristics. By mimicking natural selection processes, researchers can create molecular libraries containing vast variations of RNA sequences, allowing for the identification of variants that possess desired traits, such as increased affinity for target analytes or enhanced specificity. This approach has been particularly effective in optimizing transcription factors and riboswitches, which serve as critical components of RNA biosensors. For instance, directed evolution has been utilized to enhance the responsiveness of the PcaV allosteric transcription factor to aromatic aldehydes, leading to the development of biosensors that can effectively detect small molecules involved in microbial catabolic pathways, thereby facilitating environmental monitoring and bioremediation applications [28].

Also, in a recent study by Huo et al. [29], a riboswitch biosensor specifically responsive to a sesquiterpene amorpha-4,11-diene—was developed through SELEX-SMB that represents a biosensor and a powerful tool for the detection and regulation of small molecules, with broad applications in strain engineering, pathway regulation, and metabolic optimization in synthetic biology.

Recent advancements have highlighted the potential of utilizing large RNA aptamer libraries to generate biosensors capable of detecting diverse small molecules, extending the capabilities of RNA-based sensing technologies (Townshend et al., 2022) [30]. Townshend et al. investigated RNA-based biosensors for detecting small molecules, which is crucial for applications in synthetic biology, healthcare, and environmental monitoring. Using the DRIVER (Diversity-Rich In Vitro Evolution with RNA) method, the study enabled the multiplexed selection of RNA aptamers against unmodified small molecules, mimicking high-throughput drug screening conditions. The approach yields diverse RNA aptamers with strong binding affinities, advancing the development of genetically encoded biosensors. This work accelerates biosensor development for detecting and quantifying small metabolites in complex biological environments by demonstrating the feasibility of multiplexed selections. As the field evolves, combining computational techniques for predicting RNA structure and function alongside experimentally directed evolution will likely yield new solutions in biosensing, leading to novel diagnostic tools that can address critical needs in environmental monitoring, healthcare, and biological research.

Aptamers, which are single-stranded DNA or RNA molecules, can be isolated from combinatorial libraries using the Systematic Evolution of Ligands by Exponential Enrichment (SELEX) process. This strategy mimics natural evolutionary principles to yield high-affinity and high-specificity nucleic acid receptors, effectively serving as recognition elements in biosensors. Recent innovations in SELEX methodologies, including Capture-SELEX and integrated expression platforms, have further refined the directed evolution of nucleic acids for biosensor development. For example, Capture-SELEX techniques have been successfully employed to select DNA aptamers for aminoglycoside antibiotics by directly isolating sequences that not only bind their targets but also exhibit the signaling properties required for biosensor function (Stoltenburg et al., 2012) [31]. Similarly, strategies that integrate an expression platform into the SELEX cycle have enabled the selection of aptamers optimized for binding disease biomarkers, thereby enhancing both the functional performance and the robustness of the resulting biosensor (Ao et al., 2022) [32].

Despite these advances, challenges remain in bridging the gap between laboratory selection and real-world biosensing applications. The integration of evolved aptamers into diverse detection platforms, such as fluorescence, electrochemical, and paper-based biosensors, has demonstrated improved analytical performance; for instance, RNA aptamers have been tailored for detecting specific antibiotics, like levofloxacin, with relatively few selection cycles (Kramat et al., 2024) [33]. This study presents the development and characterization of an RNA aptamer-based biosensor for the detection of levofloxacin, a fluoroquinolone antibiotic widely used in human and veterinary medicine. The study describes the selection of a high-affinity RNA aptamer through a SELEX-based approach and its integration into a sensing platform using fluorescence quenching as a transduction mechanism. The aptamer exhibited strong specificity for levofloxacin over other structurally similar antibiotics, with a dissociation constant (Kd) in the low micromolar range, indicating good binding affinity. The sensor was capable of detecting levofloxacin concentrations in the nanomolar to micromolar range, making it suitable for applications in pharmaceutical quality control and potentially in environmental monitoring, where antibiotic contamination is a concern.

Moreover, ongoing improvements in selection techniques, including the refinement of screening conditions and amplification fidelity, are critical to overcoming practical limitations, such as high detection limits and reproducibility issues (Jaeger et al., 2019) [34]. The work presents the development of a paper-based biosensor utilizing an RNA aptamer specific to ciprofloxacin, a widely used antibiotic. The researchers characterized the binding affinity and specificity of the RNA aptamer to ciprofloxacin, confirming its potential as a biorecognition element. They employed inkjet printing technology to immobilize the aptamer onto paper substrates, creating a cost-effective and user-friendly platform for antibiotic detection. This approach offers a promising avenue for the development of portable and accessible diagnostic tools, particularly beneficial in resource-limited settings where the rapid and reliable detection of antibiotics is crucial for monitoring environmental contamination and ensuring food safety.

A summary of the biosensors improved with protein and nucleic acid engineering, as well as their characteristics, is given in Table 1.

**Table 1:** A summary of the most representative examples of biosensors improved using protein/nucleic acid engineering and their characteristics.

**Biosensor**	**Detection**	**LOD**	**Linear Range**	**Sample**	**References**
Protein engineering for biosensors					
TP2-Rho P53 peptide biosensor	R248Q mutant of protein p53	Nanomolar concentrations of p53 mutant in lung cancer cell extract	NR	Lung cancer cells	[1]
Pb whole-cell biosensor	Detection of Pb	0.045 µg/L	NR	Food products, water samples	[2]
Bacterial Cd biosensor (CadR)	Detection of Cd	0.45 µg/L	NR	Soil and water	[7]
Bacterial biosensor for detecting lead toxicity	Detection of Pb	0.012 µM Pb	0.012–3.125 µM Pb	Artificially polluted water	[8]
Bacterial biosensor for C1 molecules	Detection of formate, formaldehyde, and methanol	NR	1.0–250 mM formate 1.0–50 µM formaldehyde 5–400 mM methanol	Cell culture	[9]
Bacterial biosensor	Detection of organophosphorus compounds	10 µM parathion	NR	Artificial samples	[25]
Nucleic acid engineering for biosensors					
Riboswitch biosensor	Detection of sesquiterpene	NR	10–100 mg/mL amorpha-4,11—diene	Samples for high throughput screening and for metabolic engineering	[29]
Nucleotide aptamer	Detection of levofloxacin	100 µM	NR	Artificial samples	[33]
Nucleotide aptamer	Detection of ciprofloxacin	The maximum residue limit (MRL)	NR	Food samples	[34]

## 4. Nanomaterials Engineering for Biosensors

Nanomaterials have transformed the development of biosensors, providing distinct properties that improve biosensor performance, including enhanced stability, repeatability, and sensitivity [35]. Nanomaterials possess a high surface-area-to-volume ratio, great electronic conductivity, and excellent magnetic and physicochemical properties [36,37], and they provide enhanced accessibility for the analyte to the sensing element. Different kinds of nanomaterials, including carbon nanomaterials, metal and metal oxide nanoparticles, magnetic nanoparticles, and quantum dots, etc., have been successfully applied to develop biosensors. Numerous studies have investigated the use of different carbon nanomaterials in biosensors, demonstrating their ability to produce highly sensitive biosensors. These materials have proven to be effective transducers in electrochemical processes due to their exceptional properties, including chemical stability, high electrical conductivity, a large surface-to-volume ratio, and robust mechanical strength [38]. Carbon nanotubes, carbon nanofibers, quantum dots, graphene, and graphene oxide are commonly used carbon materials for biosensor development. The easy modification and functionalization of carbon-based nanomaterials have allowed their diverse integration with sensing elements in biotechnology [39].

For instance, Ansari et al. (see Table 2) utilized a nanocomposite consisting of two carbon nanomaterials: graphitic carbon nanosheets and carboxyl-functionalized multiwalled carbon nanotubes, which were modified with zirconium oxide nanoparticles, for the development of a biosensor [40]. They reported a label-free electrochemical aptasensor for the ultrasensitive detection of matrix metalloproteinase-9 (MMP-9), a biomarker for various pathological conditions. The proposed modification approach for carbon nanomaterials resulted in a significant increase in the electroactive surface area, by 205%, enabling the better immobilization of the matrix metalloproteinase aptamer. As a result, the sensor demonstrated an impressively broad detection range from 50 to 1250 pg/mL, with an outstandingly low limit of detection of 10.51 pg/mL. Additionally, the aptasensor exhibited real-world applicability when tested with human serum and saliva samples.

Similarly, Shi et al. conducted a study where they utilized multiwalled carbon nanotubes that were modified with gold and manganese dioxide nanoparticles to develop an electrochemical biosensor for the detection of serum homocysteine (Hcy): a reliable biomarker for cardiovascular, cerebrovascular, and neurological diseases [41]. The researchers applied the proposed composite to screen-printed carbon electrodes, resulting in the creation of a portable, disposable sensor with exceptional electrochemical activity and catalytic ability. The biosensor demonstrated a wide linear range, high sensitivity, and a low detection limit for homocysteine, making it a promising tool for the accurate determination of serum homocysteine levels. This development holds potential for enabling convenient home health monitoring for individuals at risk of these diseases.

du Plooy et al. developed a highly sensitive, label-free electrochemical immunosensor for SARS-CoV-2 IgG antibody detection through the modification of screen-printed carbon paste electrodes with graphene quantum dots (GQDs) [42]. Graphene quantum dots are a zero-dimensional (0D) member of the carbon family, consisting of a single to a few layers of graphene sheets with lateral dimensions of <10 nm [43]. GQDs possess extraordinary physicochemical characteristics, including edge effects, non-zero band gaps, and quantum confinement effects, as a result of which they hold great potential in the energy, electronic, and optical industries, as well as biosensing [44]. du Plooy et al. confirmed in their work that GQDs were instrumental in raising the detection sensitivity and expanding the active surface area of the working electrode, resulting in the improved binding efficiency of the immunocomplex. The sensing mechanism depends on the disruption of the redox reactions and electron transfer kinetics of the Fe(CN)_6_^3−/4−^ redox probe, due to the formation of an immunocomplex between the specific antigen and the IgG antibody. The high sensitivity of the sensor was confirmed by achieving a low limit of detection (LOD) of 2028 ng/mL, while remarkable selectivity was proven by the minimal interference of other antibodies. Additionally, the authors examined the possibility of using other highly conductive and low-oxygen-content carbon nanomaterials (electrochemically reduced graphene oxide and carbon quantum dots) for sensor development. Their results indicate that the performance of the electrochemical immunosensor is primarily determined by its binding affinity, rather than its electrode conductivity, which may be enhanced by utilizing carbon–carbon conjugated carbon nanostructures.

Nanostructured conducting polymers (nCPs) are receiving increasing interest, with regard to the development of biosensors, due to their biocompatibility in cellular environments [45]. Mirzaei et al. reported an aptasensor for *H. pylori* (Hsp60) detection using reduced graphene oxide (RGO) decorated with gold nanoparticles (Au NPs) [46]. The main role of the RGO was as a medium, assembling different parts of the biosensor, while the Au NPs improved the system’s conductivity and provided functional groups for aptamer attachment. In addition, the authors used a conductive polymer, PTP, in order to fill the surface of the RGO at sites where there were no Au NPs, and this method prevented the attachment of the analyte to the surface and any false responses from the biosensor. The determination of Hsp60 was studied in a hexacyanoferrate medium, and the sensitivity of the prepared sensor was investigated by several electrochemical techniques (CV, SWV, and EIS analysis) to check the effect of the used material on the performance of the sensor after each synthesis step. Their results indicate that the prepared aptasensor has suitable selectivity for Hsp60, among other biological materials.

Jaradat et al. have developed an innovative electrochemical impedimetric immunosensor that can detect *H. pylori* bacteria directly by using the HopQ protein as a biomarker [47]. The biosensor fabrication process involves electropolymerizing a layer of polypyrrole nanotubes (PPy-NTs) onto SPCE, followed by the drop-casting of multiwalled carbon nanotubes (MWCNT-COOH). The researchers chose PPy-NTs because they have excellent conductivity, biocompatibility, and redox capabilities. This simplifies the sample preparation process by eliminating the need to add redox probes during measurement. MWCNT-COOH provides covalent binding sites for HopQ antibodies (HopQ-Ab) on the biosensor surface. The results demonstrate excellent selectivity and a dynamic linear range of 5 pg/mL to 1.063 ng/mL. The calculated limit of detection was 2.06 pg/mL.

High-entropy nanomaterials have gained significant attention in research due to their unique synergy effect from multiple metals and exceptional material properties. These materials provide increased active sites, optimized electronic structures, and improved electronic conductivity in electrochemical immunoassays [48]. Lv et al. have developed an innovative, label-free electrochemical immunosensor using a self-supported, PtPdMnCoFe, high-entropy alloy with nanochain-like internetworks (HEAINN) for the highly sensitive detection of the biomarker neuron-specific enolase (NSE) [49]. The proposed material was created using a simple, one-pot, wet-chemical, co-reduction strategy and has been shown to be effective for the reduction of H_2_O_2_, acting as a signal amplifier for a novel, label-free, electrochemical, amperometric immunosensor for the highly sensitive determination of NSE. Under optimized analytical conditions, the sensor has demonstrated a broad linear range (0.1 pg mL^−1^ to 200 ng mL^−1^) and a low limit of detection (LOD = 0.0036 pg mL^−1^). Furthermore, it has successfully quantified NSE in human serum samples, yielding highly satisfactory results. Similarly, Tang et al. prepared well-defined, PtRhMoCoFe, high-entropy, alloyed nanodendrites (HEANDs), by a wet-chemical, co-reduction method and adopted it to build an electrochemical, label-free biosensor for an ultrasensitive bioassay of the biomarker cardiac troponin I (cTnI) [50]. By using the developed method, the authors achieved a low detection limit of 0.0095 pg mL^−1^ and further explored the practicability of the method in serum samples with satisfactory recovery (102.0%).

Recent breakthroughs in nanotechnology have paved the way for the development of functional nanomaterials with inherent enzyme-like activity. These innovative nanomaterials, referred to as “nanozymes”, effectively replicate the catalytic action of natural enzymes. Nanozymes demonstrate comparable kinetic behaviors to natural enzymes and effectively catalyze the conversion of substrates into oxidized products, offering promising applications in biosensing [51].

Metal–organic frameworks have great potential in developing nanozymes, due to their ability to precisely identify uniformly distributed active sites at the atomic scale in MOF-based nanozymes, offering valuable insights into catalytic mechanisms and enabling the design of more-efficient catalytic systems [52,53]. Huang et al. developed a high-performance electrochemical biosensor based on a highly active, dual-nanozyme-amplified system [54]. For this purpose, they used conductive MOF nanosheets decorated with high-density, ultrafine gold nanoparticles (Au-NPs). The MOFs provide a large surface area and abundant active, open metal sites (Cu–O_4_), which could improve the catalytic activity towards H_2_O_2_. The large number of exposed oxygen atoms also provided additional sites for the deposition of gold nanoparticles without agglomeration. The combination of the high catalytic activity of MOF and Au-NP, along with their unique structural and electrical properties, resulted in a nanohybrid-modified electrode with an excellent sensing performance for H_2_O_2_. It achieved an extremely low detection limit of 5.6 nM and a high sensitivity. Furthermore, this nanoscale electrochemical biosensor was used for the real-time monitoring of H_2_O_2_ released from different human colon cells to distinguish colon cancer cells from normal colon epithelial cells. This demonstrates great potential for the early diagnosis and management of various cancer diseases.

An innovative 3D-printed device enhanced with an iron-based metal–organic framework (Fe(II)-MOF) has been created for measuring glucose levels in artificial sweat [55]. In this study, Koukouviti et al. developed an Fe(II)-MOF that is water-insoluble and non-toxic, serving as a nanozyme for detecting glucose through differential pulse voltammetry (DPV). The electrochemical device, consisting of three electrodes and a holder, was fabricated using 3D printing technology in a single step. The Fe(II)-MOF was applied to the working electrode, followed by trapping it with a Nafion film. Unlike most glucose sensors, the DPV detection of glucose was carried out in a slightly acidic environment. The Fe(II)-MOF exhibited the capability to capture glucose on its surface and convert it to gluconolactone through the redox activity of Fe centers during the electrode’s pre-polarization step and subsequent anodic potential scan. With 3D printing allowing the creation of wearable electrochemical sensors using flexible filaments, the proposed Fe(II)-MOF/3D-printed device opens up possibilities for on-skin glucose monitoring.

A summary of the most representative examples of biosensors improved using nanomaterial engineering and their characteristics is given in Table 2.

**Table 2:** A summary of the most representative examples of biosensors improved using nanomaterial engineering and their characteristics.

Electrode Nanomaterial	Analyte	Linear Range	LOD	Real Matrices	Reference
Graphitic carbon nitride/zirconium dioxide/multiwalled carbon nanotubes	Metalloproteinase-9	50 to 1250 pg/mL	10.51 pg/mL	Human serum and saliva	[40]
Gold/manganese (IV) oxide/multiwalled carbon nanotubes	Homocysteine	5–125 μM/L	0.6173 μM/L	Serum homocysteine	[41]
Graphene-oxide-doped gold nanoparticles conjugated with polythiophene	*H. pylori*	10–900 nM/L	0.0080 μM/L with impedance; 0.0067 μM/L for SWV method	/	[46]
Polypyrrole nanotubes and carbon nanotubes nanocomposite	*H. pylori* outer membrane protein (HopQ)	5 pg/mL 1.063 ng/mL	2.06 pg/mL	Spiked drinking water	[47]
Self-supported PtPdMnCoFe	Neuron-specific enolase (NSE)	0.1 pg/mL–200 ng/mL	0.0036 pg/mL	Human serum samples	[49]
Dendritic quinary PtRhMoCoFe	cTnI	0.0001–200 ng/mL	0.0095 pg/mL	Serum samples	[50]
MOF@Pt@MOF nanozyme	Exosomal miRNA	1 fM/L–1 nM/L	0.29 fM/L	MCF-7 cells, MCF-10 A cells, and serums of a breast cancer patient and a healthy person were tested	[53]
2D conductive MOF nanosheets/gold nanoparticles	H_2_O_2_	50 nM/L–16.4 mM/L	5.6 nM/L	Human colon cells	[54]
Fe(II)-MOF nanozyme	Glucose	100–600 μmol/L	17.6 μmol/L	Artificial sweat samples	[55]

## 5. Biosensors for Rare Diseases Monitoring

### 5.1. Biosensors for the Detection of Aβ42, a Biomarker for Alzheimer’s Disease Diagnosis

Alzheimer’s disease (AD) is a slowly progressing degenerative condition with an extended initial period, during which effective therapies are lacking. Timely identification and intervention during the early phases of AD are widely acknowledged to enhance treatment outcomes. Utilizing body fluid biomarkers is a valuable approach for the prompt detection of early-stage AD. Amyloid β (1–42) (Aβ42) is a crucial biomarker for monitoring AD development. Electrochemical biosensors provide a rapid, portable, and field-capable method for analyzing Aβ42 levels, offering significant benefits in this context. Because of all of the above, a rapid increase in research on this topic has been observed.

Yu and co-authors introduced a novel approach for the highly sensitive detection of soluble β-amyloid peptides (Aβ1–40/1–42) using an electrochemical nanoprobe composed of HRP–Au–gelsolin. Instead of relying on antibody-based detection methods focusing on peptide termini recognition, the study highlights the potential of the specific interaction between gelsolin and Aβ for precise and sensitive peptide evaluation. By developing the HRP–Au–gelsolin nanohybrid through Au nanoparticle functionalization, this group of authors established a sandwich-type sensor array capturing soluble Aβ1–40/1–42 with surface-bound gelsolin and enhancing electrochemical signals via HRP catalysis. This innovative methodology demonstrated high sensitivity and a broad linear range towards Aβ1–40/1–42, achieving a remarkable detection limit of 28 pM suitable for monitoring Aβ levels in both normal and Alzheimer’s disease rat brains. The experimental results indicated significant decreases in soluble β-amyloid levels in the cerebrospinal fluid (CSF) and specific brain tissues of AD model rats compared to the normal group, showing the potential of this new method for early AD diagnosis [56].

A study conducted by Diba Sharmin and co-authors [57] showed a demonstration of an electrochemical immunosensor by using a surface sandwich complex on a gold nanoparticle (NP)-modified, screen-printed carbon electrode for the highly sensitive detection of the amyloid-beta 1–42 peptide (A) at femtomolar levels in serum and plasma. This involved the use of selective antibodies (antiA (12F4) and (1E11)) with distinct binding sites for the amyloid-beta 1–42 peptide and the formation of surface sandwich complexes of antiA (12F4)/A/antiA (1E11)-ALP employing a sequential adsorption method. The immobilized ALP enzyme, upon reaction with the substrate, 4-amino phenyl phosphate, generated voltammetric signals that increased linearly with the A concentration. The applied differential pulse voltammetry established the lowest detectable concentration of 100 fM of amyloid-beta 1–42 peptide within a linear response range from 100 fM to 25 pM. After optimization, the immunoassay platform was successfully used to determine the native concentration of the amyloid-beta 1–42 peptide in diluted human serum and plasma samples, with the results validated using a commercially available ELISA test.

A study by Ding and co-authors [58] focused on the use of the amyloid-β peptide (1–42) for the early diagnosis of Alzheimer’s disease, emphasizing its potential for high diagnostic accuracy. The researchers designed an immunosensor based on the peroxidase-like activity of heme-Aβ42 for the purpose of detecting Aβ42. Through a series of modifications to the glassy carbon electrode, including the electrodeposition of Au particles and the successive addition of polythionine–methylene blue (PTH−MB), AuNPs, monoclonal antibodies (anti-Aβ42), and bovine serum albumin (BSA), a competitive recognition capability for Aβ42 and heme-Aβ42 was achieved. The anchored heme-Aβ42 exhibited robust electrocatalytic activity in the presence of H_2_O_2_ and ferrocenemethanol. The study determined detection limits of 17.3 pM in a PBS of pH 7, 25.2 pM in serum, and 23.8 pM in saliva, with a satisfactory recovery range of 71.0% to 117.8% under optimized conditions.

A study done by Hsu and co-workers [59] reported the development of an Alzheimer’s disease biosensor for the early detection of amyloid-beta 1–42 protein by employing a simple nanorestructuring technique involving the oxidation–reduction cycle (ORC) via an electrochemical system to modify a Au sheet plate. The ORC technique was utilized to enhance the topology of Au substrates through the roughening and growth of Au grains in a KCl electrolyte solution, resulting in approximately 15% larger electroactive sites compared to polished Au without ORC. The roughened substrate was further functionalized with the specific antibody β-amyloid Aβ (1–28) through HS-PEG-NHS modification, enabling the detection of Aβ (1–42) peptide. The ORC structure provided the detection of the Aβ (1–42) peptide, with a low detection limit of 10.4 fg/mL and a wide linear range of 10^−2^ to 10^6^ pg/mL. This innovative structure demonstrated promising potential as an early-stage Alzheimer’s disease (AD) detection platform, offering low-cost fabrication and ease of operation.

A study reported by Le and co-authors [60] applied a capacitive biosensor for the non-Faradaic detection of the aβ 1–42 peptide. This approach involves the immobilization of a specific anti-aβ antibody onto a self-assembled, monolayer-functionalized, interdigitated, chain-shaped electrode. What sets this research apart from previous studies is its direct detection of the aβ peptide without the use of a redox probe ((Fe(CN)_6_)^3−^/^4−^), eliminating the potential protein denaturation caused by metallization. The non-Faradaic capacitive measurement technique employed for the direct detection of aβ without a redox probe represents a significant departure from the Faradaic measurement approach, avoiding the charge transfer resistance of the redox probe. By measuring the relative change in electrode interfacial capacitance due to specific antibody-aβ binding, the biosensor demonstrated a linear detection range of between 10 pg/mL and 104 pg/mL in human serum. The high binding affinity of the anti-aβ/SAM/ICE biosensor is evidenced by the small dissociation constant, Kd, of the antibody–antigen interaction, indicating its potential for the label-free and direct measurement of the aβ 1–42 peptide for point-of-care Alzheimer’s disease diagnosis without the need for a redox probe.

A study done by Ribeiro and co-workers [61] introduces a potentiometric biosensor designed for the point-of-care analysis of amyloid β-42 (Aβ-42). This method utilizes the molecular imprint polymer technique, where covalently immobilized Aβ-42 is utilized to create specific detection sites on the surface of single-walled carbon nanotubes. The biosensor fabrication involved binding Aβ-42 to the single-walled carbon nanotubes’ surface and imprinting it with acrylamide, N,N′-methylene-bis-acrylamide, and ammonium persulphate. The presence of imprinting sites was verified by comparing a molecular-imprint-polymer-modified surface with a negative control. The evaluation of the sensing material’s ability to rebind Aβ-42 was carried out using Raman spectroscopy and Fourier transform infrared spectroscopy, followed by an analytical performance assessment via potentiometric transduction: cationic slopes of 75 mV-decade^−1^ in a buffer of pH 8.0 and a detection limit of 0.72 μg/mL. The molecular imprint polymer material demonstrated favorable cationic slopes and a low detection limit, affirming its capability to discriminate Aβ-42 in the presence of other biomolecules in a given solution. This innovative biosensor presents a promising approach for the detection of Aβ-42, with potential applications in point-of-care analysis.

### 5.2. Biosensors for the Monitoring of Hepatitis B

Monitoring and treating hepatitis B are essential due to the potential development of acute or chronic hepatitis, which can result in serious health complications. Regular monitoring enables the early detection of infection and the timely implementation of appropriate treatment, significantly improving the disease outcome. Continuous hepatitis B treatment, including vaccination and therapy, plays a key role in suppressing the HBV virus and preventing potential hepatitis complications.

A study conducted by Chen and co-authors [62] aimed to develop an efficient, sensitive, precise, and cost-effective method for genotyping hepatitis B viruses (HBVs). This involved the use of mercapto-modified B1-, B2-, C1-, and C2-specific genotyping probes, each comprising two probes for every HBV genotype, creating a dual verification system. These probes were immobilized on four gold electrodes via Au-S bonds, allowing for the distinction of different genotypes based on the differential charge generated by RuHex binding to DNA phosphate groups before and after hybridization. The results demonstrated distinct changes in the detected charges during hybridization with genotypes B and C, as well as a significant increase in charges during hybridization with mixed genotypes (B and C) at all four electrodes. The method exhibited a wide linear detection range (10^−10^–10^−7^ M) and a high sensitivity for detecting mixed B or C HBV genotypes, proving to be a simple, specific, and cost-effective approach for rapid HBV genotyping via electrochemical sensing. This innovative method offers the sensitive detection of mixed B and C HBV genotypes, with significant potential for practical application.

An innovative, wireless-based detection system utilizing an electrochemical card (eCard) controlled by a smartphone was created to target the hepatitis B surface antigen (HBsAg) by Teengam and co-workers [63]. This label-free electrochemical platform provides user-friendly operation for convenient point-of-care diagnosis. The modification of a disposable, screen-printed carbon electrode with chitosan and glutaraldehyde, followed by antibody immobilization, established a simple, yet efficient and stable, method verified by electrochemical techniques. The smartphone-controlled eCard sensor quantified HBsAg by detecting the change in the current response of the [Fe(CN)_6_]^3−/4−^ redox couple upon HBsAg presence, achieving a linear calibration curve from 10 to 100,000 IU/mL with a detection limit of 9.55 IU/mL. Successfully tested on 500 chronically HBV-infected serum samples, the HBsAg eCard sensor exhibited high sensitivity (97.75%) and specificity (93%), highlighting its practicality and accuracy.

In a study from Fan and co-authors [64], the researchers focused on preparing amino-functionalized Ti_3_C_2_Tx MXene and fabricating a highly sensitive electrochemical DNA biosensor for the swift detection of HBV-DNA. By regulating the surface’s−NH2 concentration and anchoring gold nanoparticles, they successfully controlled the density of p-DNA, leading to the creation of a robust, stable, and specific Ti_3_C_2_NH_2_ MXene@Au nanocomposite DNA sensor. This sensor exhibited a wide detection range of 10^−17^–10^−7^ M and a low detection limit of 1.05 × 10^−14^ M, enabling the detection of HBV-DNA fragments, even in an artificial serum environment. This innovative biosensor holds promise for advancing the rapid and accurate detection of HBV-DNA, supporting the overall management and prevention of hepatitis B.

In a study by Li and co-authors [65], the research introduces a straightforward paper-based electrochemical sensor, created through paper folding, capable of detecting a 30-base nucleotide sequence specific to DNA from the hepatitis B virus with a remarkable sensitivity of 85 pM. This innovative device was developed based on previous design principles used for protein detection via a metalloimmunoassay. It boasts several key features: 1. the design integrates a simple origami assembly with a hollow-channel paper structure, ideal for accommodating micrometer-scale particles, along with a practical slip layer for precise incubation timing; 2. the sensor achieves two amplification stages: using silver nanoparticle labels for a potent amplification factor of up to 250,000 and strategically placing magnetic microbeads for an additional ~25-fold amplification at the detection electrode; 3. the assay does not require enzymes or antibodies, enhancing its speed, stability, and reliability significantly; 4. a single sample incubation step initiates the detection process, streamlining the workflow effectively.

### 5.3. Biosensors for the Monitoring of Human Papillomavirus

Monitoring for HPV is crucial because certain types of the virus, such as HPV-16, are linked to the development of cervical cancer, which can be prevented if caught at an early stage. Regular monitoring can help identify changes in cervical cells caused by HPV, allowing for timely treatment to prevent the progression to cancer. Additionally, monitoring for HPV enables healthcare providers to assess the infection status and provide appropriate care, contributing to better overall health outcomes for individuals.

Parrek and co-authors [66] constructed a new device that uses DNA to detect human papillomavirus-16 (HPV-16), which is linked to cervical cancer. To build this tool, the scientists modified a glass electrode with indium tin oxide (ITO) and added graphene oxide and silver-coated gold nanoparticles to sense HPV-16. They then attached specific DNA probes for HPV-16 to the modified electrode. Using special techniques, the scientists examined the characteristics of these materials at a microscopic level. By using various methods, like cyclic voltammetry, they analyzed how the DNA probes interacted with the target DNA by measuring electrical changes. This new biosensor displayed significant sensitivity, detecting HPV-16 effectively with a detailed range of concentrations. The successful development of this biosensor indicates a promising future for quick and accurate point-of-care diagnostic tools, potentially enabling the early detection of HPV-16 infections. The developed biosensor showed a sensitivity as high as 0.54 mA/aM with a linear range of 100 aM to 1 μM and a 100 aM LOD; the proposed biosensor exhibited an excellent performance with a rapid diagnosis.

A study conducted by Yang and co-workers [67] introduced a unique biosensor by combining a modified gold electrode and a super sandwich structure using 3-aminopropyltriethoxysilane (APTES), resulting in a biosensor with a lower detection limit (5.475 × 10^−16^ mol/L) and a wide linear range (1.0 × 10^−13^ mol/L to 1.0 × 10^−6^ mol/L). The experimental results demonstrated the biosensor’s good stability, and further testing revealed its potential as a reliable clinical tool for disease diagnosis with strong interference resistance in complex human serum samples. This innovative biosensor has promising implications for the early detection and management of cervical cancer.

The development of an electrochemical sensor for detecting HPV 16 by using dual-signal amplification is a significant step in improving the early diagnosis and prevention of cervical cancer. This kind of sensor utilized an APTES-modified glassy carbon electrode to enhance its stability and incorporated gold nanoparticles and a chain amplification reaction for signal amplification [68]. With a limit of detection (LOD) of 1.731 × 10^−16^ mol/L and a wide linear response range, the sensor demonstrated great potential, exhibiting the good recovery of serum samples and strong anti-interference properties. The emphasis on low cost, high sensitivity, and stability aligns with the future prospects of developing effective cervical cancer prevention methods and electrochemical biosensors.

A study done by Rasouli and co-authors [69] presents a label-free biosensor designed to detect HPV-16 using a straightforward and environmentally friendly method. The screen-printed carbon electrodes were coated with Fe_3_O_4_-Au core-shell nanoparticles to enhance their sensing capabilities. Then, the electrode surfaces were functionalized with thiolated single-strand DNA (ssDNA) probes specific to HPV DNA sequences. The hybridization process was monitored using cyclic voltammetry and differential pulse voltammetry with [Fe(CN)_6_]^3−^/^4−^ as the redox indicator, successfully distinguishing the absence and presence of immobilized probe DNA. The biosensor demonstrated its optimal performance at a probe DNA concentration of 5 μM, achieving a limit of detection of 0.1 nM and a sensitivity of 2.4 μA/nM, providing a promising avenue for the development of effective HPV detection tools.

A very interesting study was done by Chaibun and his co-workers [70]. In contrast to previous studies, where the focus was on the detection of a specific analyte—HPV 16 peptide—in this study with a sandwich assay, the concentration of two types of analytes—HPV 16 and HPV 18 peptides—was determined at the femtomolar detection limit. The method utilizes a sandwich hybridization process involving the HPV target, a silica-redox dye reporter probe, and a capture probe, with subsequent electrochemical detection. This biosensor exhibits high specificity and sensitivity, detecting HPV-16 at a limit of 22 fM and HPV-18 at 20 fM, within a wide range of 1 fM to 1 µM. Assessment using oral and cervical samples demonstrated the biosensor’s consistency with nested PCR/gel electrophoresis detection. Its quick completion time of 90 min, simplicity, and high sensitivity make it suitable for HPV detection in clinical laboratories and epidemiological studies, offering an alternative to existing detection methods. This innovative biosensor holds great promise for efficient HPV detection.

A summary of the techniques and biomarkers used in some recent research articles to improve biosensors is presented in Table 3.

## 6. Conclusions

The engineering of proteins, nucleic acids, and nanomaterials plays a key role in the development of advanced biosensors that enable the early detection and monitoring of rare diseases. Using innovative engineering techniques, including protein evolution, aptamer design, and nanomaterial engineering, these biosensors offer high specificity and sensitivity in detecting the specific biomarkers of rare diseases, even at very low concentrations, which is vital for timely intervention, thereby significantly improving treatment, while at the same time reducing the cost and simplifying the treatment of the disease itself. The individual or group integration of these technologies into wearable devices opens new opportunities for continuous patient monitoring, advancing personalized medicine and improving treatment outcomes. Currently, these systems are still in the development and research phase, but their application could have a significant impact on the future of rare disease diagnostics, providing healthcare professionals with better tools for assessing and managing patient conditions. The importance of this research is also reflected in the increased number of papers in this area, some of which are summarized in this review.

Proteins and nucleic acids offer several distinct advantages over nanomaterials in the development of biosensors, particularly in terms of biorecognition, specificity, and compatibility with biological systems. Engineered proteins, such as enzymes, transcription factors, and antibodies, exhibit high catalytic efficiency and target selectivity, enabling the sensitive detection of specific analytes under physiological conditions. Nucleic-acid-based elements, including aptamers and DNAzymes, provide programmable and highly tunable recognition capabilities through predictable base-pair interactions, allowing the design of versatile and robust sensing platforms. Unlike many synthetic nanomaterials, which may require complex functionalization and raise concerns about biocompatibility or environmental toxicity, biomolecules are inherently biocompatible. They can be sustainably produced via recombinant or synthetic methods. Furthermore, their molecular adaptability supports integration into various transduction systems, enhancing the modularity and precision of biosensor designs.

From a commercial perspective, integrating engineered proteins, nucleic acids, and nanomaterials into biosensors presents promising avenues for developing scalable, cost-effective, and highly sensitive diagnostic platforms. Advances in synthetic biology and nanotechnology have accelerated the development of the next-generation biosensors suitable for real-time, point-of-care, and wearable applications. With a growing market demand across healthcare, environmental monitoring, and food safety, these bioengineered systems will drive innovation and commercialization, bridging the gap between laboratory research and practical deployment.

In the coming years, further intensive work in this field is expected, expanding research capacities, and further progress in reducing costs, improving accuracy, and expanding the availability of these technologies is expected, contributing to the global improvement of healthcare and the quality of life of patients with rare diseases.

## Figures and Tables

**Table 3:** Summary of techniques and biomarkers used in recent biosensor research.

Field	Technique	Biosensor Type	Biomarkers	Advancements	Refs.
Protein Engineering	Directed evolution	Optical, Electrochemical	Mutant p53, Pb, Cd, C1 compounds, cholesterol, transcription factors	High-specificity biosensors via protein mutation and screening	[1,2,3,4,5,6,7,8,9,10]
	Semi-rational design	Optical, Electrochemical	Glucarate, D-serine, D-alanine, metabolites, acephate	Enhanced enzyme selectivity/stability using structural data	[11,12,13,14,15,16,17]
	Rational design	Optical, Electrochemical	Estrogen, lactate, calcium, ATP, NAD+, HPP, pesticides	Targeted mutations for ligand binding, allosteric sensor design	[18,19,20,21,22,23,24,25]
Nucleic Acid Engineering	SELEX and aptamer engineering	Fluorescent, Electrochemical	Aminoglycosides, levofloxacin	Improved aptamer affinity and specificity for antibiotics and proteins	[26,27,31,32,33]
	Riboswitch development	Fluorescent	Amorpha-4,11-diene, aromatic aldehydes	Riboswitches for metabolite detection in synthetic biology	[28,29]
	RNA aptamer screening (DRIVER)	Fluorescent, Paper-based	Small molecules	High-throughput aptamer generation with multiplex selection	[30]
Nanomaterials Engineering	Carbon nanomaterials (CNTs, graphene)	Electrochemical	MMP-9, *H. pylori*, SARS-CoV-2 IgG	Enhanced conductivity and immobilization for pathogen detection	[38,40,41,42,46,47]
	MOF-based nanozymes	Electrochemical	Exosomal miRNA, H_2_O_2_, glucose	Ultrasensitive catalytic detection via MOF-Au composites	[52,53,54,55]
	High-entropy alloy nanomaterials	Electrochemical	NSE, cTnI	Multi-metallic platforms with ultralow LOD for disease markers	[48,49,50]
